# High-Speed Imaging of Second-Harmonic Generation in MoS_2_ Bilayer under Femtosecond Laser Ablation

**DOI:** 10.3390/nano11071786

**Published:** 2021-07-09

**Authors:** Young Chul Kim, Hoseong Yoo, Van Tu Nguyen, Soonil Lee, Ji-Yong Park, Yeong Hwan Ahn

**Affiliations:** 1 Department of Physics and Department of Energy Systems Research, Ajou University, Suwon 16499, Korea; zerofe@ajou.ac.kr (Y.C.K.); kgod3645@gmail.com (H.Y.); tunv@ajou.ac.kr (V.T.N.); soonil@ajou.ac.kr (S.L.); jiyong@ajou.ac.kr (J.-Y.P.); 2 Institute of Materials Science, Vietnam Academy of Science and Technology, Hanoi 100000, Vietnam

**Keywords:** second-harmonic generation, transition-metal dichalcogenides, twisted bilayer, laser ablation

## Abstract

We report an in situ characterization of transition-metal dichalcogenide (TMD) monolayers and twisted bilayers using a high-speed second-harmonic generation (SHG) imaging technique. High-frequency laser modulation and galvano scanning in the SHG imaging enabled a rapid identification of the crystallinity in the TMD, including the orientation and homogeneity with a speed of 1 frame/s. For a twisted bilayer MoS_2_, we studied the SHG peak intensity and angles as a function of the twist angle under a strong interlayer coupling. In addition, rapid SHG imaging can be used to visualize laser-induced ablation of monolayer and bilayer MoS_2_ in situ under illumination by a strong femtosecond laser. Importantly, we observed a characteristic threshold behavior; the ablation process occurred for a very short time duration once the preheating condition was reached. We investigated the laser thinning of the bilayer MoS_2_ with different twist angles. When the twist angle was 0°, the SHG decreased by approximately one-fourth of the initial intensity when one layer was removed. Conversely, when the twist angle was approximately 60° (the SHG intensity was suppressed), the SHG increased abruptly close to that of the nearby monolayer when one layer was removed. Precise layer-by-layer control was possible because of the unique threshold behavior of the laser-induced ablation.

## 1. Introduction

The noncentrosymmetric structures of transition-metal dichalcogenides (TMDs) enable an efficient second-harmonic generation (SHG). Hence, TMDs are advantageous in the development of next-generation optoelectronic devices with the increasing demand for smaller and thinner nonlinear media [1,2,3,4,5,6,7,8,9,10,11]. The SHG provides information on the crystal orientation, degree of crystallization, and thickness and stacking sequence of the TMD structure, which is useful for nondestructive crystal structure studies [3,4,5,6,7,8,9,10,11,12,13,14,15,16,17]. For example, 2H-phase molybdenum disulfide (MoS_2_) with an odd number of layers retains the second-order nonlinear signal generation due to its broken inversion symmetry. Conversely, in MoS_2_ with an even number of layers, the restoration of the crystal inversion symmetry largely suppresses the SHG signals [6,7,8,9,10,11]. Recently, twisted TMD layers have attracted particular interest since the twisted angle serves as a novel degree of freedom to control their unique electronic and optical characteristics [18,19,20,21,22,23,24]. SHG signals from artificially stacked TMD bilayers exhibited intensity and polarization variations as a function of the twisted angle between the two layers [8,12,25]. In addition, polarization-resolved SHG techniques enabled the determination of the relative orientation [12,17,25].

In order to achieve SHG signals from the monolayer and few-layer TMDs, a femtosecond laser was focused through a microscope in which the SHG intensity was recorded as a function of the laser polarization angle. On the other hand, point-by-point imaging with SHG signals is a very powerful method for mapping of grain boundaries, homogeneity, structure-induced nonlinear enhancement, and interfaces between different layers [9,10,11,13,14,15,16]. Although transmission electron microscopy provides an accurate crystal orientation and high resolution, it requires complex procedures for sample preparation. Conversely, optical microscopy does not provide sufficient intrinsic contrast for differentiation of grain boundaries within the layer [26]. In addition, it is essential to provide a measurement time below a couple of seconds for practical applications of the potential in-line inspection of the crystallinity of TMDs, including their orientation. For example, in situ identification of crystallinity will be very useful for finding the optimal conditions of chemical vapor deposition (CVD) and post-processing, such as the laser-induced ablation of TMDs [27,28,29,30,31,32,33]. The conventional technique based on sample scanning requires a duration of a couple of minutes per frame, which results in longer measurement times to achieve the full angle-resolved information. On the contrary, the galvano scanning system could provide a small SHG signal acquisition time of a few seconds per frame [26].

In this study, we introduce a high-speed scanning SHG microscopy technique by incorporating galvano scanning combined with high-frequency laser modulation. Images were acquired with a typical scanning speed of 1 frame/s and a signal-to-noise ratio (SNR) of 30 dB. This enabled a rapid identification of the crystallographic direction in TMD materials as well as the grain boundaries and homogeneity of the crystallinity. In particular, we investigated the SHG intensity of an artificially stacked bilayer MoS_2_ as a function of the twist angle. In addition, we monitored the in situ ablation of monolayer and bilayer MoS_2_ induced by the femtosecond laser.

## 2. Materials and Methods

### 2.1. Material Synthesis

MoS_2_ flakes were grown on a SiO_2_/Si substrate by atmospheric-pressure CVD with MoO_3_ (99.95%, Sigma-Aldrich, St. Louis, MO, USA) and S (99.98%, Sigma-Aldrich) powders as precursors. The substrate was placed facing down toward MoO_3_ (3 mg) on a ceramic boat, which was loaded into the center of the quartz tube in the CVD setup. A second ceramic boat containing 500 mg of sulfur powder was kept at the outside and upstream parts of the furnace, where the temperature could be controlled independently by another heating controller. The furnace and ceramic boat with the sulfur powder were heated to 650 and 200 °C with ramping rates of 32 and 10 °C/min, respectively. After 10 min of growth, the furnace was turned off and cooled to room temperature. Conversely, WSe_2_ flakes were grown on a SiO_2_/Si substrate by atmospheric-pressure CVD with WO_3_ (99.95%, Sigma-Aldrich) and Se (99.5%, Sigma-Aldrich) powders as precursors. The furnace and ceramic boat with the selenide powder were heated to 850 and 250 °C, respectively. After 10 min of growth, the furnace was switched off and cooled to room temperature.

A twisted MoS_2_ was prepared by wet transfer of the CVD-synthesized MoS_2_ flakes into another SiO_2_/Si substrate containing the MoS_2_ flakes. We used poly(methyl methacrylate) (PMMA) as a mechanical supporter, which was removed in acetone after the transfer. As there are hundreds of individual flakes on the substrate, each transfer creates dozens of bilayer heterostructures with various twist angles. Finally, the as-transferred MoS_2_ heterostructure was annealed in a vacuum environment at 400 °C for 1 h to remove PMMA residues.

### 2.2. SHG Imaging Setup

**Figure 1:**
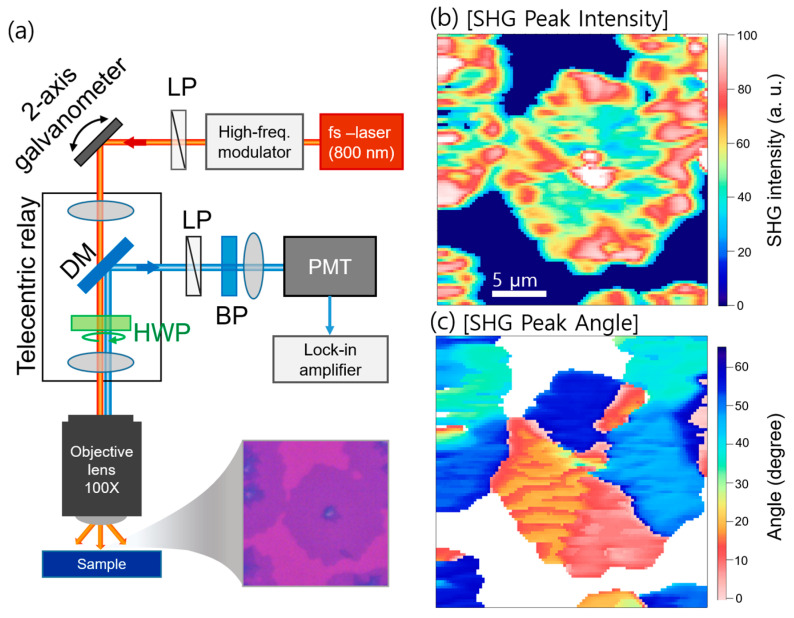
(**a**) Schematic of the SHG measurements (LP: linear polarizer, DM: dichroic mirror, HWP: zero-order half-wave plate, BP: band-pass filter, PMT: photomultiplier tube); (**b**) SHG peak intensity mapping of the WSe_2_ flakes shown in the inset of (**a**), which is extracted from the angle-dependent 2D SHG images; (**c**) Mapping of the SHG peak angle for identifying different crystal orientations within the flakes.

The rapid SHG imaging setup is illustrated in Figure 1a. A homemade femtosecond laser centered at 800 nm (with a pulse width of 30 fs and repetition rate of 80 MHz) illuminates TMD materials through an objective lens (100×, numerical aperture = 0.8) with a diffraction-limited laser focal spot (full width at half maximum (FWHM) of 650 nm) (Supplementary Materials Figure S1). In order to overcome the low scanning speed of the conventional SHG imaging, we used a two-axis galvanometer scanning mirror (GVS002, Thorlabs Inc., Newton, NJ, USA) incorporated with high-frequency laser modulation at 100 kHz (BOM, Boston Micromachines Corp., Cambridge, MA, USA), which simultaneously enabled rapid scanning and improved SNR [34,35]. The SHG signals were measured using a photomultiplier tube (H8249-101, Hamamatsu, Hamamatsu City, Shizuoka, Japan) and lock-in amplifier (AMETEK, Inc., Oak Ridge, TN, USA). The typical dwell time (and the time constant of the lock-in amplifier) was less than 0.5 ms per pixel, which allowed us to obtain the images of 1 frame/sec for the images with 50 × 50 pixels. In order to obtain the angular distribution information, we acquired the SHG image and stacked them as a function of the polarization angle of the incident femtosecond laser using a half-wave plate (HWP) mounted on a motorized rotation stage. Typically, we varied the polarization angle in the range of 0–72° with a step size of 2°. This is sufficient for identifying the intensity and peak polarization angles in TMD materials possessing six-fold symmetry (e.g., MoS_2_ and WSe_2_). We confirm that the SHG signals from WSe_2_ and MoS_2_ layers exhibited the six-fold symmetry as a function of polarization angle and a quadratic laser-power-dependence (Supplementary Materials Figure S2).

The SHG data were fitted as a function of the angle for each pixel to obtain two-dimensional (2D) images of the intensity and peak angle, as shown in Figure 1b,c, respectively. The images were acquired on monolayer WSe_2_, synthesized by CVD. The SHG was normally strong near the edge of the hexagonal shape, with the exception of the strong SHG signal in the middle, as shown in Figure 1b. Notably, in the angular distribution images in Figure 1c, we can identify different crystallization domains with direction angles of 0° to 60°, which cannot be identified in the bright-field optical microscopy images, as shown in Figure 1a. We could obtain angle-resolved images with a measurement time below 1 min. Therefore, our technique is very useful for the rapid identification of crystallization orientation, including its homogeneity.

## 3. Results and Discussion

**Figure 2:**
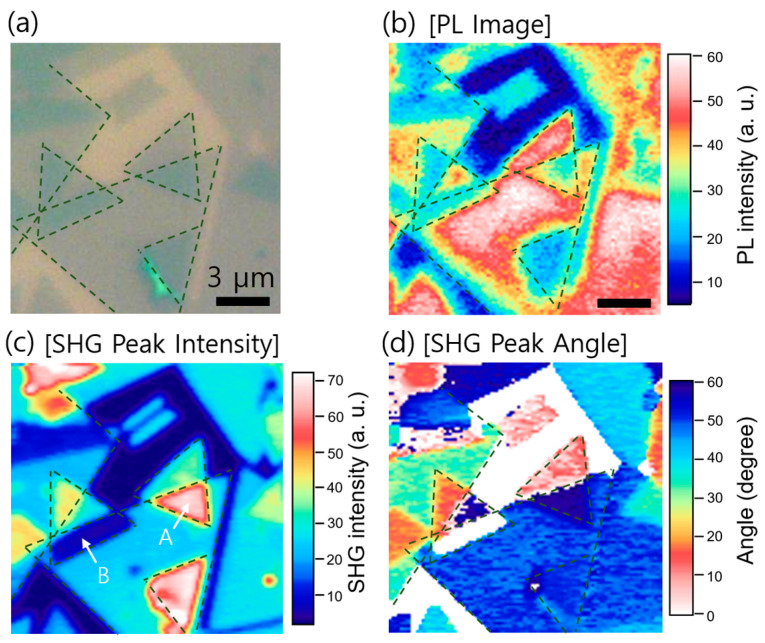
(**a**) Bright-field image of the artificially stacked MoS_2_ on the SiO_2_/Si substrate; (**b**) PL image of the MoS_2_/MoS_2_ heterostructure sample shown in (**a**). The PL quenching in the overlapped regions indicates interlayer coupling of the twisted bilayer; (**c**) SHG peak intensity mapping of the twisted bilayer MoS_2_. The SHG peak signal increases (region denoted by A) or decreases (region denoted by B) depending on the stacking angle; (**d**) mapping of the SHG peak angle for the bilayer MoS_2_.

We applied our technique to study the bilayer MoS_2_ with a random orientation, fabricated artificially by stacking the CVD-grown monolayer MoS_2_, as shown in the microscopy image in Figure 2a. The electronic and optical characteristics depending on the twist angle between the crystal orientations have attracted particular interest. The intensity of the artificially stacked MoS_2_ monolayer has been addressed and analyzed successfully in terms of polarization interference [25]. In this study, we performed a rapid imaging on many stacked MoS_2_ layers possessing a strong interlayer coupling to investigate the possibility of the SHG enhancement effects deviating from the polarization interference model. We first show a photoluminescence (PL) image for comparison in Figure 2b. PL images were taken through a monochromator fixed at the peak spectral position (at 655 nm) while raster-scanning the focused CW laser with a wavelength of 532 nm (Supplementary Materials Figure S3). The PL signal is suppressed significantly regardless of the twist angle in which spatial overlap occurred between the layers. This confirms that the interlayer coupling should be strong in the twisted bilayer because PL quenching does not occur when there is a gap between the layers, typically owing to the residues between the layers [21,36].

As shown in Figure 2c, the SHG peak intensity changes mainly in the twisted bilayer region; it increases (region denoted by A) or decreases (region denoted by B) depending on the twist angle. The twist angle information can be obtained by *θ*_twist_ = *φ*_1_ – *φ*_2_ using the angles *φ*_1_ and *φ*_2_ recorded for the adjacent layers in the image in Figure 2c. In addition, we could identify the SHG peak angle in the overlapped region, as shown in Figure 2d. The SHG was increased for *θ*_twist_ = 0°, whereas it was suppressed virtually to zero for *θ*_twist_ = 60°.

**Figure 3:**
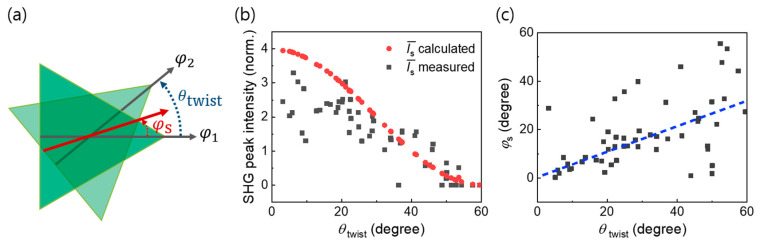
(**a**) Illustration of the angle of the twisted bilayer of MoS_2_ flakes. *φ*_1_ and *φ*_2_ indicate the armchair directions of the MoS_2_ flakes, while *φ*_s_ indicates the peak angle of the SHG emission. The twist angle is defined by *θ*_twist_ = *φ*_1_ − *φ*_2_; (**b**) normalized SHG peak intensity for the twisted bilayer MoS_2_ as a function of *θ*_twist_ (black squares). The red circles indicate the calculated SHG peak intensities derived from the polarization interference model; (**c**) SHG peak angle (*φ*_s_) at the twisted region as a function of *θ*_twist_. The dashed line corresponds to the line of *φ*_s_ = *θ*_twist_/2.

We tested more than 50 twisted bilayer MoS_2_ structures and studied the SHG peak intensity and angle as a function of *θ*_twist_ (Figure 3a); the results are presented in Figure 3b,c. The black squares in Figure 3b represent the measured SHG intensity, whereas the red circles represent values estimated by the relation $I_{s}\left(\theta_{\mathrm{twist}}\right)=I_{1}+I_{2}+2\sqrt{I_{1}I_{2}}\cos3\theta_{\mathrm{twist}}$, where $I_{1}$ and $I_{2}$ are the SHG peak intensities of the individual flakes and $I_{s}$ is the SHG peak intensity in the bilayer region. Here, we plotted normalized intensity ($\bar{I_{s}}$) using the averaged SHG peak intensities ($\left(I_{1}+I_{2}\right)/2$) of adjacent layers. Our experimental results are consistent with those expected from the polarization interference model. We could not observe extraordinary signals, for example, higher than four times that of the monolayer even when there was a strong coupling between the layers, as confirmed by the PL measurements. This is contrary to the behavior of the twisted bilayer graphene, which exhibits a large enhancement in the third-harmonic generation [37]. In addition, the peak angle (*φ*_s_) in the twisted region exhibits a value that follows the relation *φ*_s_ = (*φ*_1_ – *φ*_2_)/2, as indicated by the dashed line in Figure 3c. The relatively large deviation for *θ*_twist_ > 40° is likely due to errors originating from weak signals, which originates from the destructive interference.

**Figure 4:**
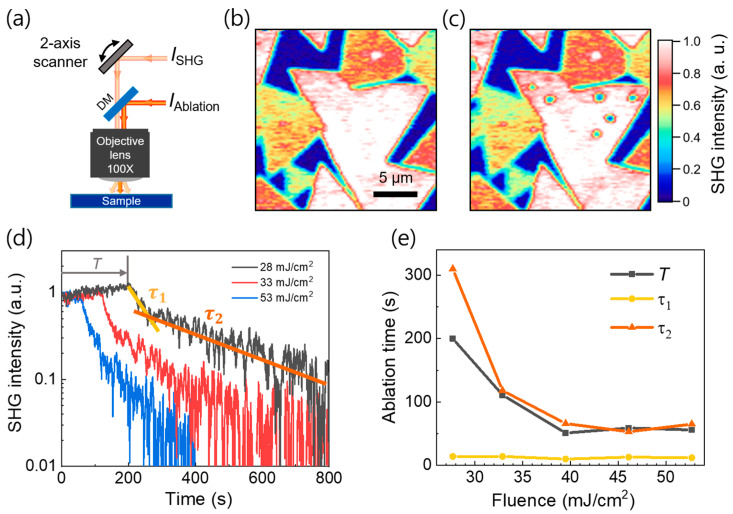
(**a**) Schematic of the in situ SHG imaging of the laser ablation; SHG intensity maps at the fixed polarization angle (**b**) before and (**c**) after the laser-induced ablation; (**d**) SHG intensity as a function of time for different laser fluences, when the focused femtosecond laser irradiated the sample at *t* = 0; (**e**) Preliminary heating time (*T*) and ablation time constants (*τ*_1_ and *τ*_2_) as functions of the laser fluence, where *τ*_1_ and *τ*_2_ are extracted by fitting the data in (d) with a biexponential function.

The rapid SHG imaging can be used to visualize laser-induced ablation in situ under illumination by a strong femtosecond laser. As the energy of the laser (1.55 eV) is lower than the MoS_2_ bandgap (1.8 eV), a two-photon process is required for ablation. The ablation by the femtosecond laser is advantageous over those by continuous-wave or nanosecond lasers in general because it results in minimal collateral damage within the sample [38]. We began with the in situ SHG imaging of the laser ablation in the monolayer MoS_2_, as shown in Figure 4. A schematic of the experimental setup for laser ablation is shown in Figure 4a. An additional femtosecond pulse was focused at a fixed position on the MoS_2_ layer, while the SHG was monitored in situ using the galvano scanning method, as discussed above.

We show the SHG intensities at a fixed polarization angle (*φ* = 27°) before (Figure 4b) and after (Figure 4c) the laser ablation when we exposed the ablation laser at six different positions (indicated by blue dots) under different laser fluence from 28 to 53 mJ/cm^2^ for 800 s. Using the transient SHG images, we present the SHG as a function of time in Figure 4d for different laser fluences used for the ablation. Notably, there is a preliminary heating time *T*, which is likely due to the heat tolerance in MoS_2_ [39,40] followed by an exponential decrease that varies with the fluence. For example, *T* of approximately 3 min was required until the ablation at *F* = 28 mJ/cm^2^. The gradual increase in the SHG signal reflects the increase in the lattice temperature because it is known that SHG increases with temperature [41].

On the other hand, the SHG exhibited a biexponential decay with time (with time constants of *τ*_1_ and *τ*_2_) once the ablation occurred. By fitting the curves, we summarize *T* and the time constants as functions of the laser fluence in Figure 4e, which provides useful information for optimization of the process. Notably, the preliminary heating time was strongly dependent on the laser fluence, whereas the decay time constant *τ*_1_ (~10 s) did not vary with the laser fluence. In other words, the laser ablation of the MoS_2_ layer occurred for a very short period of time once the condition for chemical bond breakage was reached. This is characteristic threshold behavior, which has not been reported. It is likely that the lowering of the damage threshold due to the accumulated lattice heating caused the abrupt ablation processes, whereas the detailed mechanism during the ablation processes has to be addressed further [31]. Conversely, the second decay process (with the time constant *τ*_2_) is likely due to the removal of the residues or the additional ablation around the edge of the spot (see Supplementary Materials Figure S4).

**Figure 5:**
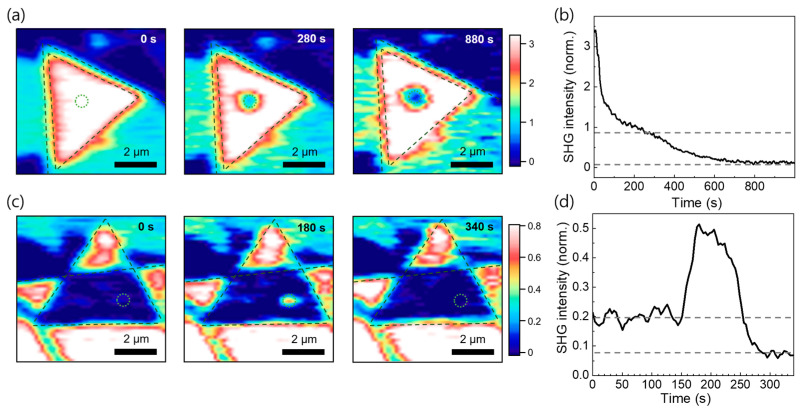
(**a**) SHG maps of the twisted bilayer with constructive interference (i.e., with *θ*_twist_ = 0) at different times (*t* = 0, 280, 880 s). The ablation laser was switched on at *t* = 0 and focused on the dashed circular area; (**b**) SHG intensity vs. time at the ablation laser spot position in (**a**). The intermediate status was observed owing to the layer-by-layer etching; (**c**) SHG maps for the twisted bilayer with destructive interference (i.e., with *θ*_twist_ close to 60˚) at different times (*t* = 0, 180, 340 s); (**d**) SHG vs. time for the laser irradiation at the laser spot position in (**c**). The abrupt increase in the SHG signal indicates that the top layer has been removed by the ablation.

Finally, we monitored the thinning of the bilayer using the rapid SHG imaging technique, as shown in Figure 5. We show a series of SHG images of a bilayer with a constructive-interference homojunction (i.e., with *θ*_twist_ = 0°) in Figure 5a (see also the full movie in Supplementary Video S1). The fluence of the ablation laser was 46 mJ/cm^2^. The SHG images acquired at *t* = 0, 280, and 880 s represent the times before ablation, when the top layer was removed, and when both layers were removed, respectively. This is more clearly shown in Figure 5b as a plot of the SHG as a function of time, where we can identify the intermediate status with the removed upper layer. In other words, the intensity is one-fourth of the initial value as shown as the dashed line, which confirms that a single layer was left and this is consistent with the polarization interference model [25].

In situ ablation images of the heterojunction with destructive interference are shown in Figure 5c,d. The SHG signal was initially suppressed because the twist angle was close to *θ*_twist_ = 60°. Conversely, a strong signal appeared at the laser spot position at *t* = 180 s that is almost as strong as that of the nearby monolayer, which disappeared at *t* = 340 s (see the full movie in Supplementary Video S2). The dynamical ablation behavior is more clearly reflected by the plot of the SHG intensity as a function of time in Figure 5d. The SHG signal was suppressed until it increased abruptly at *t* > 150 s and then disappeared at *t* > 250 s. This is a strong indication that the monolayer was formed in the middle of the ablation with the removed upper layer. Notably, the intermediate status persisted for a relatively long period (*t* = 170–250 s) due to the unique threshold behavior shown in Figure 4. This enables a precise layer-by-layer control of the twisted MoS_2_ bilayers and can help determine the optimal conditions for material processing for future electronic and optoelectronic device applications.

## 4. Conclusions

We reported the characterization of TMD bilayers through the rapid SHG imaging technique for the in situ identification of the crystallinity in the TMD, including the orientation and homogeneity. For the twisted bilayer MoS_2_, we studied the SHG peak intensity and peak angles as a function of the twist angle under a strong interlayer coupling confirmed by PL measurements. In addition, we studied the femtosecond ablation of the bilayer MoS_2_ in situ, which helped determine the optimal conditions for laser processing of the TMDs. We observed a characteristic threshold behavior: chemical bond breakage occurred for a very short time duration (~10 s) regardless of the presence of the ablation laser once the preheating condition was reached. We also investigated the thinning of the bilayer MoS_2_ for different twist angles. In the case of the homojunction, the SHG decreased by approximately one-fourth of the initial intensity when the upper layer was removed. Conversely, in the case of the heterojunction with the marginal SHG signal, the SHG increased abruptly and reached that of the monolayer when the upper layer was removed. Precise layer-by-layer control was possible due to the threshold behavior in the laser ablation. Therefore, our approach will be very useful for the in-line inspection of crystallinity in various TMDs and other functional nanomaterials and the determination of optimal conditions for their processing in the fabrication of optoelectronic devices.

## Data Availability

Data are contained within the article.

## Supplementary Materials

The following are available online at https://www.mdpi.com/article/10.3390/nano11071786/s1, Figure S1: The spatial resolution of SHG imaging, Figure S2: The polarization angle and power dependence, Figure S3: PL Spectra, Figure S4: The spatial extent of the laser-ablated area as a function of time, Video S1: Transient SHG image of a bilayer with a homojunction, Video S2: Transient SHG image of a bilayer with a heterojunction.

## References

[1] Wang G., Marie X., Gerber I., Amand T., Lagarde D., Bouet L., Vidal M., Balocchi A., Urbaszek B. (2015). Giant Enhancement of the Optical Second-Harmonic Emission of WSe_2_ Monolayers by Laser Excitation at Exciton Resonances. Phys. Rev. Lett..

[2] Kim S., Fröch J.E., Gardner A., Li C., Aharonovich I., Solntsev A.S. (2019). Second-harmonic generation in multilayer hexagonal boron nitride flakes. Opt. Lett..

[3] Seyler K.L., Schaibley J.R., Gong P., Rivera P., Jones A.M., Wu S., Yan J., Mandrus D.G., Yao W., Xu X. (2015). Electrical control of second-harmonic generation in a WSe_2_ monolayer transistor. Nat. Nanotechnol..

[4] Shi J., Yu P., Liu F., He P., Wang R., Qin L., Zhou J., Li X., Zhou J., Sui X. (2017). 3R MoS_2_ with broken inversion symmetry: A promising ultrathin nonlinear optical device. Adv. Mater..

[5] Wang Y., Xiao J., Yang S., Wang Y., Zhang X. (2019). Second harmonic generation spectroscopy on two-dimensional materials [Invited]. Opt. Mater. Express.

[6] Säynätjoki A., Karvonen L., Rostami H., Autere A., Mehravar S., Lombardo A., Norwood R.A., Hasan T., Peyghambarian N., Lipsanen H. (2017). Ultra-strong nonlinear optical processes and trigonal warping in MoS_2_ layers. Nat. Commun..

[7] Li Y., Rao Y., Mak K.F., You Y., Wang S., Dean C.R., Heinz T.F. (2013). Probing Symmetry Properties of Few-Layer MoS2 and h-BN by Optical Second-Harmonic Generation. Nano Lett..

[8] Wen X., Gong Z., Li D. (2019). Nonlinear optics of two-dimensional transition metal dichalcogenides. InfoMat.

[9] Malard L.M., Alencar T.V., Barboza A.P.M., Mak K.F., De Paula A.M. (2013). Observation of intense second harmonic generation from MoS_2_ atomic crystals. Phys. Rev. B.

[10] Zhao M., Ye Z., Suzuki R., Ye Y., Zhu H., Xiao J., Wang Y., Iwasa Y., Zhang X. (2016). Atomically phase-matched second-harmonic generation in a 2D crystal. Light. Sci. Appl..

[11] Kumar N., Najmaei S., Cui Q., Ceballos F., Ajayan P.M., Lou J., Zhao H. (2013). Second harmonic microscopy of monolayer MoS_2_. Phys. Rev. B.

[12] Psilodimitrakopoulos S., Mouchliadis L., Paradisanos I., Kourmoulakis G., Lemonis A., Kioseoglou G., Stratakis E. (2019). Twist Angle mapping in layered WS2 by Polarization-Resolved Second Harmonic Generation. Sci. Rep..

[13] Karvonen L., Säynätjoki A., Huttunen M.J., Autere A., Amirsolaimani B., Li S., Norwood R.A., Peyghambarian N., Lipsanen H., Eda G. (2017). Rapid visualization of grain boundaries in monolayer MoS2 by multiphoton microscopy. Nat. Commun..

[14] Li D., Wei C., Song J., Huang X., Wang F., Liu K., Xiong W., Hong X., Cui B., Feng A. (2019). Anisotropic Enhancement of Second-Harmonic Generation in Monolayer and Bilayer MoS_2_ by Integrating with TiO_2_ Nanowires. Nano Lett..

[15] Rosa H.G., Ho Y.W., Verzhbitskiy I., Rodrigues M.J.D.L.F., Taniguchi T., Watanabe K., Eda G., Pereira V.M., Gomes J.C.V. (2018). Characterization of the second- and third-harmonic optical susceptibilities of atomically thin tungsten diselenide. Sci. Rep..

[16] Yin X., Ye Z., Chenet D.A., Ye Y., O’Brien K., Hone J.C., Zhang X. (2014). Edge Nonlinear Optics on a MoS_2_ Atomic Monolayer. Science.

[17] Qian Q., Zu R., Ji Q., Jung G.S., Zhang K., Zhang Y., Buehler M.J., Kong J., Gopalan V., Huang S. (2020). Chirality-Dependent Second Harmonic Generation of MoS_2_ Nanoscroll with Enhanced Efficiency. ACS Nano.

[18] van der Zande A.M., Kunstmann J., Chernikov A., Chenet D.A., You Y., Zhang X., Huang P.Y., Berkelbach T.C., Wang L., Zhang F. (2014). Tailoring the Electronic Structure in Bilayer Molybdenum Disulfide via Interlayer Twist. Nano Lett..

[19] Kim K., Coh S., Tan L.Z., Regan W., Yuk J.M., Chatterjee E., Crommie M.F., Cohen M.L., Louie S.G., Zettl A. (2012). Raman Spectroscopy Study of Rotated Double-Layer Graphene: Misorientation-Angle Dependence of Electronic Structure. Phys. Rev. Lett..

[20] Kou L., Frauenheim T., Chen C. (2013). Nanoscale Multilayer Transition-Metal Dichalcogenide Heterostructures: Band Gap Modulation by Interfacial Strain and Spontaneous Polarization. J. Phys. Chem. Lett..

[21] Huang S., Ling X., Liang L., Kong J., Terrones H., Meunier V., Dresselhaus M.S. (2014). Probing the interlayer coupling of twisted bilayer MoS_2_ using photoluminescence spectroscopy. Nano Lett..

[22] Alexeev E.M., Catanzaro A., Skrypka O.V., Nayak P.K., Ahn S., Pak S., Lee J., Sohn J.I., Novoselov K.S., Shin H.S. (2017). Imaging of Interlayer Coupling in van der Waals Heterostructures Using a Bright-Field Optical Microscope. Nano Lett..

[23] Nayak P.K., Horbatenko Y., Ahn S., Kim G., Lee J.-U., Ma K.Y., Jang A.-R., Lim H., Kim D., Ryu S. (2017). Probing Evolution of Twist-Angle-Dependent Interlayer Excitons in MoSe_2_/WSe_2_ van der Waals Heterostructures. ACS Nano.

[24] Jiang T., Liu H., Huang D., Zhang S., Li Y., Gong X., Shen Y.-R., Liu W.-T., Wu S. (2014). Valley and band structure engineering of folded MoS_2_ bilayers. Nat. Nanotechnol..

[25] Hsu W.-T., Zhao Z.-A., Li L.-J., Chen C.-H., Chiu M.-H., Chang P.-S., Chou Y.-C., Chang W.-H. (2014). Second Harmonic Generation from Artificially Stacked Transition Metal Dichalcogenide Twisted Bilayers. ACS Nano.

[26] Psilodimitrakopoulos S., Mouchliadis L., Paradisanos I., Lemonis A., Kioseoglou G., Stratakis E. (2018). Ultrahigh-resolution nonlinear optical imaging of the armchair orientation in 2D transition metal dichalcogenides. Light. Sci. Appl..

[27] Xue H., Wu G., Zhao B., Wang D., Wu X., Hu Z. (2020). High-Temperature In Situ Investigation of Chemical Vapor Deposition to Reveal Growth Mechanisms of Monolayer Molybdenum Disulfide. ACS Appl. Electron. Mater..

[28] Wang Y., Zhang L., Su C., Xiao H., Lv S., Zhang F., Sui Q., Jia L., Jiang M. (2019). Direct Observation of Monolayer MoS_2_ Prepared by CVD Using In-Situ Differential Reflectance Spectroscopy. Nanomaterials.

[29] López-Posadas C.B., Wei Y., Shen W., Kahr D., Hohage M., Sun L. (2019). Direct observation of the CVD growth of monolayer MoS_2_ using in situ optical spectroscopy. Beilstein J. Nanotechnol..

[30] Su B.-W., Zhang X.-L., Xin W., Guo H.-W., Zhang Y.-Z., Liu Z.-B., Tian J.-G. (2021). Laser-assisted two dimensional material electronic and optoelectronic devices. J. Mater. Chem. C.

[31] Wang M., Li D., Liu K., Guo Q., Wang S., Li X. (2020). Nonlinear Optical Imaging, Precise Layer Thinning, and Phase Engineering in MoTe2 with Femtosecond Laser. ACS Nano.

[32] Xinghua L., Shan X., Wu Y., Zhao J., Lu X. (2017). Laser Thinning and Patterning of MoS_2_ with Layer-by-Layer Precision. Sci. Rep..

[33] Paradisanos I., Kymakis E., Fotakis C., Kioseoglou G., Stratakis E. (2014). Intense femtosecond photoexcitation of bulk and monolayer MoS2. Appl. Phys. Lett..

[34] Park J., Son B., Park J.-Y., Lee S., Ahn Y. (2013). High-speed scanning photocurrent imaging techniques on nanoscale devices. Curr. Appl. Phys..

[35] Son B.H., Park J.-K., Hong J.T., Park J.-Y., Lee S., Ahn Y.H. (2014). Imaging Ultrafast Carrier Transport in Nanoscale Field-Effect Transistors. ACS Nano.

[36] Yuan J., Najmaei S., Zhang Z., Zhang J., Lei S., Ajayan P.M., Yakobson B.I., Lou J. (2015). Photoluminescence Quenching and Charge Transfer in Artificial Heterostacks of Monolayer Transition Metal Dichalcogenides and Few-Layer Black Phosphorus. ACS Nano.

[37] Ha S., Park N.H., Kim H., Shin J., Choi J., Park S., Moon J.-Y., Chae K., Jung J., Lee J.-H. (2021). Enhanced third-harmonic generation by manipulating the twist angle of bilayer graphene. Light Sci. Appl..

[38] Kakkava E., Romito M., Conkey D.B., Loterie D., Stankovic K.M., Moser C., Psaltis D. (2019). Selective femtosecond laser ablation via two-photon fluorescence imaging through a multimode fiber. Biomed. Opt. Express.

[39] Kim H.-J., Kim D., Jung S., Bae M.-H., Yi S.N., Watanabe K., Taniguchi T., Chang S.K., Ha D.H. (2018). Homogeneity and tolerance to heat of monolayer MoS_2_ on SiO_2_ and h-BN. RSC Adv..

[40] Kim H.-J., Yun Y.J., Yi S.N., Chang S.K., Ha D.H. (2020). Changes in the Photoluminescence of Monolayer and Bilayer Molybdenum Disulfide during Laser Irradiation. ACS Omega.

[41] Khan A.R., Liu B., Zhang L., Zhu Y., He X., Zhang L., Lü T., Lu Y. (2020). Extraordinary Temperature Dependent Second Harmonic Generation in Atomically Thin Layers of Transition-Metal Dichalcogenides. Adv. Opt. Mater..

