# Supplementary material for: High-Speed Imaging of Second-Harmonic Generation in MoS2 Bilayer under Femtosecond Laser Ablation
Source: Nanomaterials (Basel). 2021 Jul 9;11(7):1786. doi: 10.3390/nano11071786 (PMC8308356; doi:10.3390/nano11071786)
Supplement: Supplementary file 1 [file nanomaterials-11-01786-s001.zip › nanomaterials-1280471 (1) PDF si rev.pdf]

*nanomaterials*

*Supplementary Materials*

# High-Speed Imaging of Second-Harmonic Generation in $MoS_2$ Bilayer under Femtosecond Laser Ablation

**Young Chul Kim [1], Hoseong Yoo [1], Van Tu Nguyen [1,2], Soonil Lee [1], Ji-Yong Park [1] and Yeong Hwan Ahn [1,*]**

[1] Department of Physics and Department of Energy Systems Research, Ajou University, Suwon 16499, Korea; zerofe@ajou.ac.kr (Y.C.K. ); kgod3645@gmail.com (H.Y.); tunv@ajou.ac.kr (V.T.N.); soonil@ajou.ac.kr (S.L.); jiyong@ajou.ac.kr (J.-Y.P.)

[2] Institute of Materials Science, Vietnam Academy of Science and Technology, Hanoi, 100000, Vietnam;

* Correspondence: ahny@ajou.ac.kr

## S1. The spatial resolution of SHG imaging

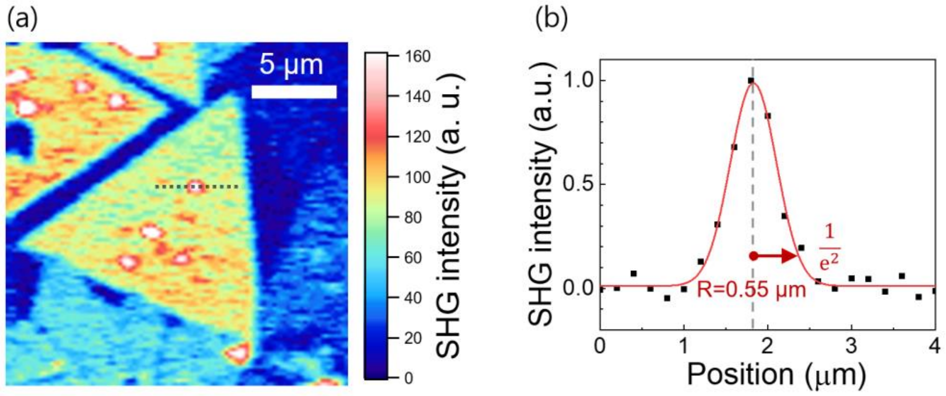


**Figure S1.** (**a**) SHG intensity maps of monolayer $MoS_2$ at fixed polarization angle. (**b**) SHG intensity taken along the dashed line shown in (**a**). The resolution (R) of the SHG signal was measured at 550 nm (full width at half maximum of 650 nm).

## S2. The polarization angle and power dependence

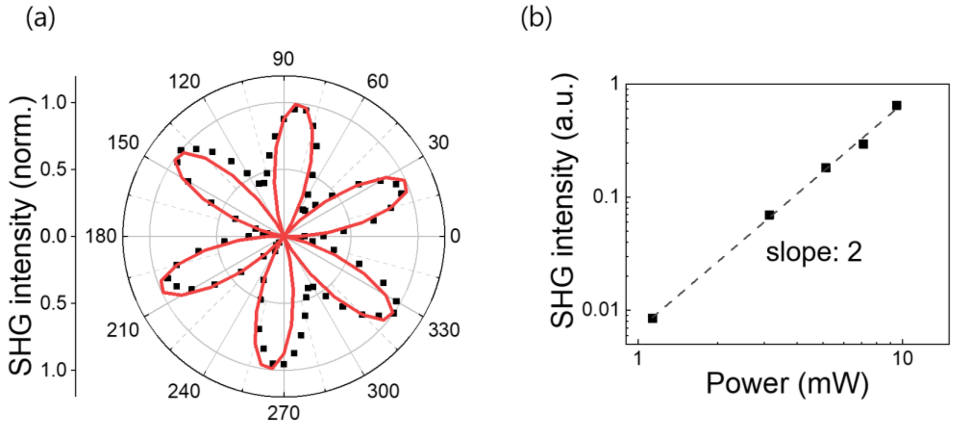


**Figure S2.** (**a**) A representative plot of SHG intensity vs polarization angle for a monolayer $MoS_2$. (**b**) SHG intensity of monolayer $MoS_2$ as a function of laser power, which exhibits a quadratic power dependence.

## S3. PL Spectra

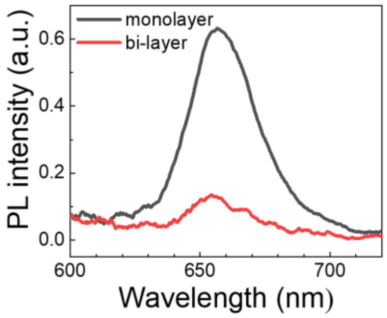


**Figure S3.** Representative photoluminescence (PL) spectra of monolayer (black) and bilayer (red) $MoS_2$ as shown in the Figure 2b of the main text. The PL spectra were peaked at 655 nm for both monolayer and bilayer $MoS_2$.

## S4. The spatial extent of the laser-ablated area as a function of time

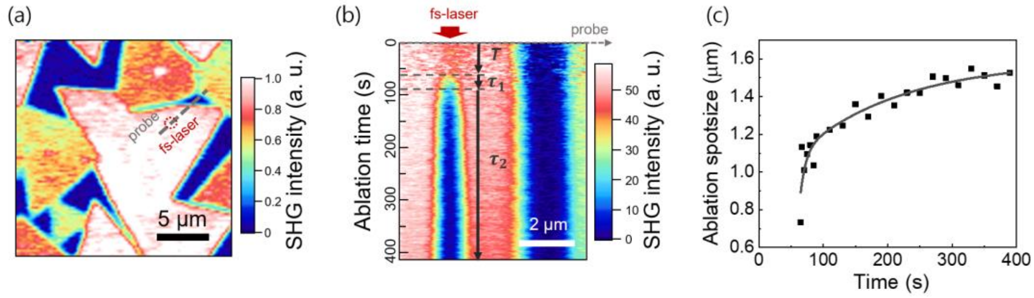


**Figure S4.** (**a**) SHG image for a monolayer $MoS_2$. (**b**) SHG intensity as a function of the position along the monolayer $MoS_2$ (x-axis) and ablation time (y-axis) along the dashed line in (**a**). A focused fs-laser for ablation was located in the circular area in (**a**) with a fluence of 53 mJ/cm$^2$. The preliminary heating time $T$ was 60 s and decay time constants of $\tau_1$ and $\tau_2$ were 16 s and 74 s, respectively. (**c**) The ablated spot size was plotted as a function of time extracted from (b), which exhibited similar bi-exponential decay with time constants of $\tau_1$=6 s and $\tau_2$=112 s.
